# Novel variation in the *CEL* gene causing impaired fasting glucose in a Chinese pediatric patient: case report and literature review

**DOI:** 10.3389/fendo.2026.1706262

**Published:** 2026-01-23

**Authors:** Chang Su, Yurong Piao, Congli Chen, Di Wu, Rongmin Li, Yanmei Sang

**Affiliations:** 1 Department of Pediatric Endocrinology, Genetics, and Metabolism, Beijing Children’s Hospital, Capital Medical University, National Center for Children’s Health, Beijing, China; 2 Department of Immunology, Beijing Children’s Hospital, Capital Medical University, National Center for Children’s Health, Beijing, China; 3 Department of Pediatrics, West China Second University Hospital, Sichuan University, Chengdu, China; 4 Key Laboratory of Birth Defects and Related Diseases of Women and Children, Ministry of Education, West China Second University Hospital, Sichuan University, Chengdu, China; 5 Department of Endocrinology, Beijing Children’s Hospital Affiliated to Capital Medical University Baoding Hospital, Baoding, China

**Keywords:** carboxyl ester lipase (CEL), CEL-MODY, diabetes mellitus, gene mutation, impaired fasting glucose, pancreatic exocrine dysfunction

## Abstract

**Objective:**

CEL-related Maturity-Onset Diabetes of the Young (CEL-MODY) is a rare form caused by carboxyl ester lipase (*CEL*) gene mutations. It is characterized by dysglycemia and pancreatic exocrine dysfunction. We described a case to highlights the heterogeneity of clinical manifestations of the *CEL* gene mutations in pediatric patients.

**Case presentation:**

We report a 12-year-old boy presenting with impaired fasting glucose. The patient reported no abdominal pain. Magnetic resonance imaging (MRI) of the pancreas revealed no evidence of pancreatic atrophy, fatty infiltration, or other abnormalities. Additionally, the fecal elastase level was within the normal range. Genetic analysis identified a novel heterozygous mutation in the *CEL* gene (c.1809dupC). The child exhibited only early-stage diabetes without concomitant pancreatic exocrine insufficiency, indicating a phenotypically mild form.

**Conclusion:**

Children with *CEL* gene mutations appear to exhibit significant phenotypic heterogeneity. It may be correlated with both the specific mutation type and age at disease onset. Thus, lifelong, systematic monitoring of pancreatic endocrine and exocrine function is clinically necessary.

## Introduction

Maturity-Onset Diabetes of the Young (MODY) is a group of autosomal dominant monogenic diabetes mellitus (DM) accounting for approximately 1–5% of all DM cases (1). It is characterized by early-onset (ranging from 6 months to 25 years), absence of autoimmune-antibodies, non-insulin-dependent. CEL-related Maturity-Onset Diabetes of the Young (CEL-MODY, OMIM#609812) is an autosomal dominant inherited subtype characterized by co-existence of dysglycemia and impaired pancreatic exocrine function. It demonstrated that CEL-MODY is caused by single-bp deletions in the proximal variable number of tandem repeats (VNTR) segments in the *CEL* gene (2). CEL-MODY is extremely rare, as only five families and a case have been described. To date, CEL-MODY has not been reported in China. Furthermore, relatively few cases have been reported in children.

CEL-MODY was first reported by Ræder et al. in two Norwegian families (3). In family 1, 33 patients carried a single-base deletion mutation (c.1686delT) in the *CEL* gene. Fourteen patients had DM, and three had impaired glucose tolerance. All mutation carriers exhibited moderate-to-severe decreases in fecal elastase levels. Ten patients had pancreatic atrophy revealed in a CT scan, and one presented with pancreatic fibrosis in pathological examination at the age of 46. Because the age at onset was less than 25 years in 3 diabetic cases, the family met the criteria for maturity-onset diabetes of the young (MODY), categorizing as MODY8 or CEL-MODY. In family 2, four cases revealed a single-base deletion (1785delC) in the fourth VNTR region. Three patients had DM, and one mutation carrier maintained normoglycemia. Moderate-to-severe fecal elastase decrease was observed in all mutation carriers.

Previous studies demonstrated the heterogeneity of the *CEL* gene mutations and the variability of its phenotypic manifestations. Several pathogenic mutations in the *CEL* gene have been identified, but their clinical manifestations vary considerably. Previous studies have identified new pathogenic or likely pathogenic mutations in the *CEL* gene in patients with type 2 diabetes mellitus (T2DM), and MODY-X who lacked pancreatic exocrine dysfunction (4–6). On the other hand, Helge Ræder et al. reported 11 nondiabetic children with mutations in the *CEL* gene. The patients exhibited decreases in fecal elastase levels. Pancreatic magnetic resonance imaging (MRI) findings indicated pancreatic steatosis (7).

Herein, we report a novel heterozygous frameshift mutation (c.1809dupC) in the proximal VNTR region of the *CEL* gene in a Chinese boy presenting with impaired fasting glucose. We hypothesize that children with *CEL* gene mutations may exhibit a mild clinical phenotype. Furthermore, our study provides a comprehensive review to enhance the understanding of the clinical and genetic manifestation characteristics of CEL-MODY.

## Case report

A 12-year-old boy was admitted to our hospital because of persistent abnormal blood glucose. Given that the child’s mother was diagnosed with DM, the child was further tested for glycated hemoglobin (HbA1c), which was found to be slightly elevated. Retrospectively, during the preoperative examination for adenoid surgery when the child was 11 years old, a fasting blood glucose level of 6.15 mmol/L (reference range: 3.9–5.9 mmol/L) was detected. He had no history of polyphagia, polydipsia, polyuria, or weight loss. Body mass index (BMI) was 18.8 kg/m^2^(z score 0.4). He showed no facial abnormalities, acanthosis nigricans, buffalo back, or purple stripe on skin. He was the first child of his non-consanguineous parents (G1P1).

Results of the oral glucose tolerance test (OGTT) showed that fasting plasma glucose (FPG) was 6.11mmol/L(3.9-6.1mmol/L), C-peptide was 2.83ng/mL(1.1-5.0 ng/mL), and insulin level was 16.1 μIU/mL(6.0-27 μIU/mL). 2h blood glucose was 5.84mmol/L(<7.8mmol/L), C-peptide was 5.37ng/mL(2.8-7.0 ng/mL), and insulin was 25.4μIU/mL(13.2-44.1 μIU/mL). HbA1c level was 6%(3.8-5.8%). The levels of triglycerides and cholesterol are normal. Islet cell antibodies (ICAs), insulin autoantibodies (IAAs), glutamic acid decarboxylase autoantibodies (GADAs), and anti-tyrosine phosphatase-like insulinoma antigen 2 (anti-IA2) antibodies were negative. Hormones (triiodothyronine, thyroxine, thyroid stimulating hormone, cortisol levels, prolactin, testosterone, parathyroid hormone) were all normal. Abdominal ultrasound and Magnetic resonance imaging (MRI) showed the structure and morphology of the pancreas is normal. The level of fecal elastase was normal.

His mother was diagnosed with DM at the age of 36. His father has no history of diabetes. Her results of the oral glucose tolerance test (OGTT) showed that fasting plasma glucose (FPG) was 4.77mmol/L(3.9-6.1mmol/L), C-peptide was 1.16ng/mL(1.1-5.0 ng/mL), and insulin level was 2.67μIU/mL(6.0-27 μIU/mL). 2h blood glucose was 15.1mmol/L(<7.8mmol/L), C-peptide was 8.98ng/mL(2.8-7.0 ng/mL), and insulin was 49.2μIU/mL(13.2-44.1 μIU/mL). HbA1c level was 6.7%(3.8-5.8%).

**Figure 1:**
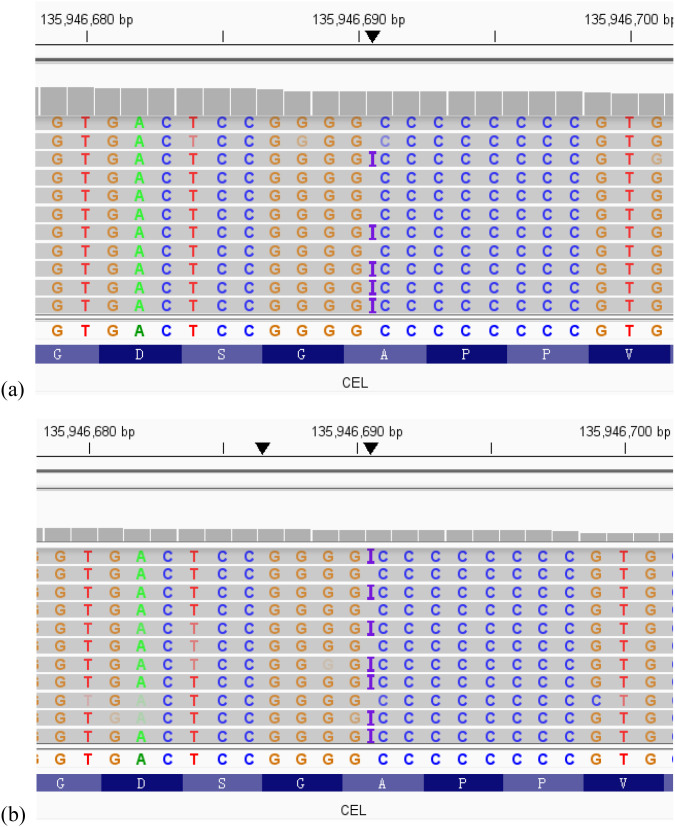
The visualization results from the Integrative Genomics Viewer (IGV) of whole-exome sequencing (WES) showed that the child **(a)** and the child’s mother **(b)** carried the same novel heterozygous variant in the *CEL* gene.

Informed parental consent was obtained before genetic investigations. Whole exome sequencing (WES) revealed a heterozygous mutation, c.1809dup, in exon11 of the *CEL* gene. This mutation resulted in a frameshift mutation at the amino acid level (p. Val604ArgfsTer6), which changes valine at position 604 to arginine, completely alters the subsequent amino acid sequence, and leads to premature termination of the protein after only 5 additional abnormal amino acids are added. This mutation is novel and absent in the population databases. According to the American College of Medical Genetics and Genomics (ACMG) guidelines, this variant was classified as likely pathogenic (PVS1_Moderate+PS4+PM2_Supporting). Sanger sequencing validation failed due to homologous sequence interference and high GC content in the VNTR region. As previously reported in studies, this high-GC environment impairs DNA polymerase activity during PCR and frequently leads to 3’-end stuttering in sequencing results (8). We therefore performed next-generation sequencing (NGS) on the child’s parents. The results showed that the child’s mother carried the same mutation in the *CEL* gene (**Figure 1**).

The only treatment for the child is dietary control. The mother’s treatment consists of dietary control combined with oral administration of acarbose. The mother discontinued acarbose after 4 months. During follow up both the child and the mother have achieved good blood glucose control.

## Discussion

We report a 12-year-old boy who presented with impaired fasting glucose, which were early indications of prediabetes. The signs of pancreatic atrophy or fatty infiltration were not observed by ultrasound and MRI. Our patient’s mother was diagnosed with DM at the age of 36. Genetic analysis revealed our patient, and his mother carried the same novel frameshift mutation in VNTR region of the *CEL* gene. The patient carrying *CEL* gene mutation exhibited mildly abnormal blood glucose levels without pancreatic exocrine insufficiency. This suggests that clinical phenotypes associated with *CEL* gene mutations are heterogeneous.

The human *CEL* gene is located on chromosome band 9q34 and contains 11 exons. VNTR region is in exon 11 and characterized by a guanine/cytosine-rich repeat unit consisting of 33-base pair (bp) sequences. The repetition counts of the VNTR region vary in humans, ranging from 7 to 21 times, mostly 16 times. The *CEL* gene encodes the carboxyl ester lipase, which plays a crucial role in the metabolism of cholesterol and fats. The CEL protein is approximately 11 kb and is mainly expressed in pancreatic acinar cells. The structure of the CEL protein includes a signal peptide, a globular domain containing bile salt binding sites and catalytic sites, and a C-terminal segment (9). The coding region of the signal peptide is located in exon 1, and the catalytic sites are in exons 5, 8, and 10, respectively. The coding region of the C-terminal segment is the VNTR region. The C-terminal segment is comprised of 11 amino acid repeats, which include amino acids (proline [P], glutamate [E], aspartate [D], serine [S], and threonine [T]) (10). It is enriched with O-glycosylation sites that are critical for ensuring the protein’s correct folding, secretion, and stability.

It has elucidated CEL-MODY is a protein misfolding disease due to disfunction of O-glycosylation sites in VNTR region (11). Abnormal carboxyl ester lipase can lead to pancreatic exocrine dysfunction. Earlier studies demonstrated that the mutant CEL proteins overaccumulated both intracellularly and extracellularly. β-cells internalize the mutant protein via endocytosis, leading to intracellular aggregates. As a result, the affected β-cells exhibited the downregulation of the *GCK* and *ABCC8* genes, thereby reducing glucose sensitivity (12).

We identified a novel frameshift mutation c.1809dupC located in exon 11 (VNTR region). This mutation causes premature termination of amino acid transcription, thereby impairing the protein structure. Our study found that due to the presence of a large number of repetitive sequences in the VNTR region, combined with interference from homologous sequences and excessively high GC content, Sanger sequencing validation failed repeatedly. Thus, we performed next-generation sequencing (NGS) for the child’s mother to verify the mutation. Although validation was successfully achieved, the testing cost remains relatively high. Therefore, exploring the application of simple and low-cost sequencing methods for detecting mutations in the VNTR region could be a direction for further research in the future.

Typical clinical characteristics of CEL-MODY included abnormalities of glucose metabolism and pancreatic exocrine function. In the two Norwegian kindreds, mean age at diagnosis of diabetes (years) was 34 ± 12. The BMI is around 24 ± 2.9. HbA1C range to 8.5 ± 1.4. Fasting glucose range to 10 ± 4.1 mmol/l. Some mutation carriers exhibit early sign of DM such as impaired fasting glucose or abnormal glucose tolerance. One mutation carrier previously reported remained nondiabetic until the age of 42 (8). About 97.9% patients display signs of pancreatic exocrine dysfunction. The exocrine function of the pancreas tends to be affected mainly after the second decade. Patients mainly present with recurrent abdominal pain or steatorrhea. Fecal elastase levels are all decreased, and MRI may reveal signs of pancreatic atrophy, steatosis, and cysts. Mild abdominal pain may precede morphological changes in the pancreas (7). In a Swedish family report, the brother of the proband, who was diagnosed with acute pancreatitis with very low fecal elastase at the age of 45 years, was found to have recurrent abdominal pain since the age of 13 years by retrospective history. Additionally, some patients (10/15) with *CEL* gene mutation presented with demyelinating peripheral neuropathy, which is not related to the course and severity of DM, suggesting that demyelinating peripheral neuropathy may be another rare clinical feature of CEL-MODY (13).

**Figure 2:**
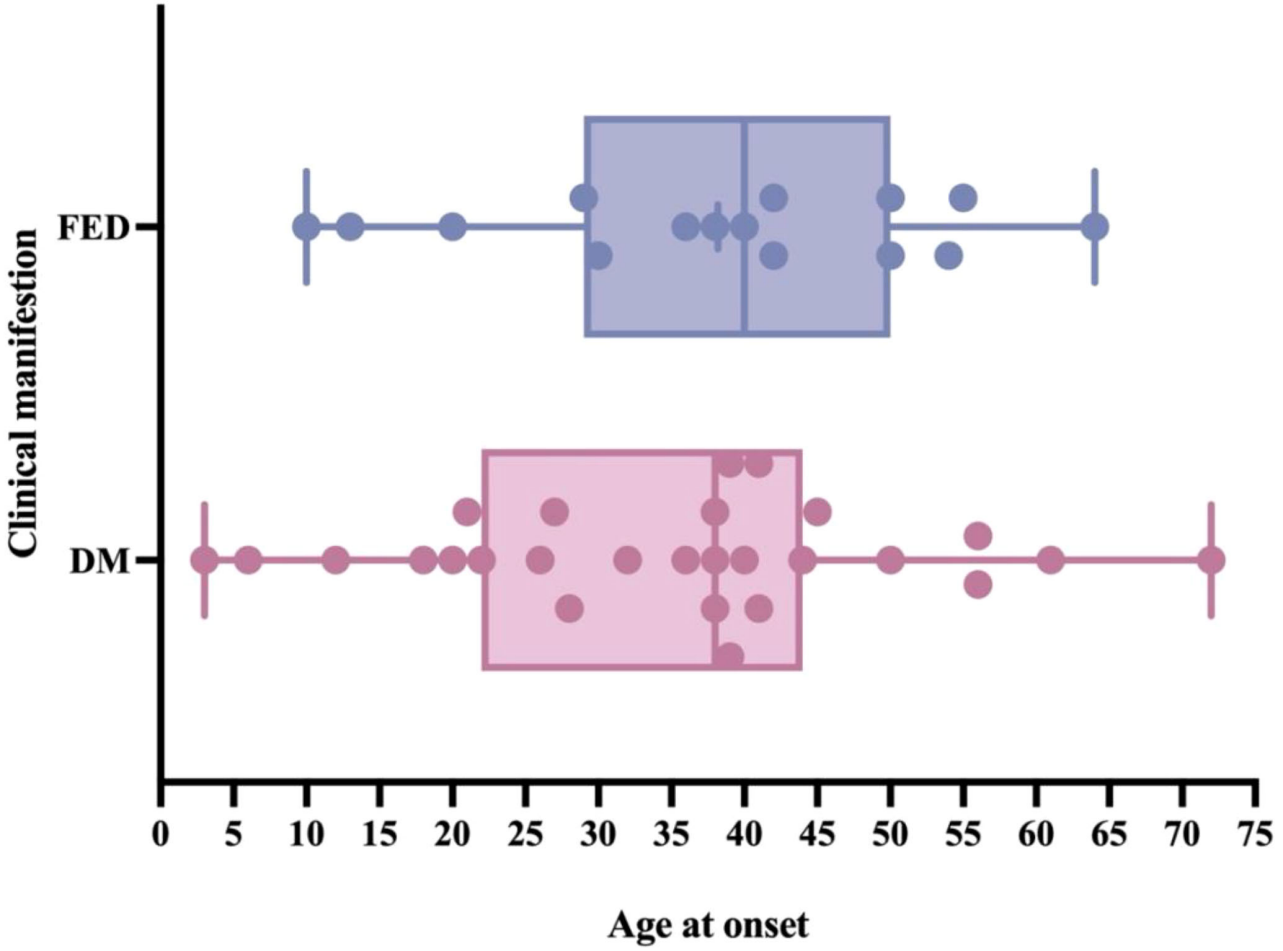
The onset age of diabetes mellitus (DM) and fecal elastase deficiency (FED) in CEL-MODY patients. Only 2 pediatric cases reported to date, and 86.7% of patients developed FED after the age of 20. Pancreatic exocrine dysfunction appears to manifest later than diabetes.

In this study, the patient had an onset age of 11 years. The main manifestation was an incidental finding of elevated fasting blood glucose, with no signs of pancreatic exocrine gland damage. Based on the clinical manifestations, a diagnosis of CEL-MODY cannot be established at present. This child presents with a mild clinical phenotype caused by the *CEL* gene mutation. Regarding the putative reasons, it may be related to the age at disease onset. We summarized the CEL-MODY patients’ onset age of DM and pancreatic exocrine dysfunction (**Figure 2**). Only 2 pediatric cases reported to date, and 86.7% of patients developed fecal elastase-1 deficiency (FED) after the age of 20. A key clinical observation is that in these patients, pancreatic exocrine dysfunction manifests later than diabetes. These results imply that the lack of pancreatic manifestations in prior cases might be related to their age at assessment (5, 6, 14). The age may be associated with the clinical phenotype for *CEL* gene mutation carriers, highlighting the need for long-term follow-up of individuals carrying such mutations.

Additionally, earlier studies indicate that mutation type and location of the *CEL* gene may play a critical role in multiple phenotypes. Overall, more than 80 identified mutations in the *CEL* gene have been documented in the HGMD database. The mutation type mainly included missense, nonsense, deletions, frameshift, and splice-site mutations. Khadija et al. concluded that only single-bp deletions in the VNTR region are associated with CEL-MODY (2). Most *CEL* mutations, such as missense mutations, do not cause CEL-MODY. Previous CEL-MODY reports show that mutations all occur in VNTR segments. A single base-pair deletion (c.1686delT and 1785delC) in the VNTR region of the *CEL* gene was firstly identified in two Norwegian families in 2006 (3). In 2010, a novel CEL allele was identified in a Danish CEL-MODY family by multiplex PCR and fragment analysis, which contained only 3 VNTR repeats—an abnormally low copy number that deviates significantly from the normal range of VNTR repeats in the general population (8). In 2021, a 38-year-old Italian patient of CEL-MODY was reported (15). Gene analysis showed a heterozygous mutation (c.1811delC) in the 5th VNTR region of the *CEL* gene, resulting in a frameshift mutation that shortened the VNTR region. The study in 2022 identified the c.1685delC mutation in the VNTR region in the Swedish family and the c.1786delG mutation in the VNTR region in the Czech family, respectively (2). Anny Gravdal (16) et al. demonstrated that the longest aberrant protein tails, resulting from single-base deletions in the proximal VNTR region, have the highest pathogenic potential.

Many studies have identified a variety of pathogenic or probable pathogenic mutations not located in the VNTR region of the gene. Huixiao Wu (17)et al. identified five novel *CEL* gene mutations in a diabetes cohort study. The mutation sites were Ex.8-11del, c.830G>T (p. Cys277Phe), c. 2193_2225del (p. Gly729_Thr739del), Ex.10-11del, c.1621C>T (p. Arg540Cys). Protein function prediction software showed that all could affect protein function. Two mutations, p. Gly729_Thr739del and p. Arg540Cys, were subjected to *in vitro* functional experiments, which showed that CEL expression was reduced in HEK293 cells transfected with the mutant genes and additionally resulted in intracellular retention of the mutant proteins. A case report in Japan revealed c.146_147delCT mutation in exon 2 in the *CEL* gene in a 13-year-old child without decrease of fecal elastase levels (6). Additionally, In 2024, Siyu Sun et al. identified p. Val736Cysfs*22 mutation affecting the 16th VNTR in the *CEL* gene in two patients with early-onset T2DM. Pancreatic exocrine dysfunction was not observed as the mutation didn’t lead to a new tail region of the enzyme (18). The other two patients carrying deletion mutations (p. Thr684ArgfsTer9, p. Pro740fs) did not present pancreatic exocrine dysfunction either (19, 20). In this study, our patient’s clinical phenotype may be related to the gene mutation site. Regrettably, due to limitations in our conditions, we are unable to verify the length of the VNTR region resulting from the impact of the gene mutation in this child.

Regarding the treatment of CEL-MODY, pancreatic enzyme replacement therapy is recommended for patients with pancreatic exocrine dysfunction. The dosages are delineated as follows: infants should be administered 2,000-4,000 U lipase/kg/meal per 120 mL of formula, whereas children(age> one year) should be administered 500-2,500 U lipase/kg/meal for meals and 250-1,000U lipase/kg/meal for snacks (21). The management of DM in patients with CEL-MODY may involve insulin therapy or oral antidiabetic agents. It was mentioned that approximately 23.8% of the CEL-MODY cases were treated exclusively with oral antidiabetic agents, 66.7% with insulin, and 9.5% with a combination of both oral antidiabetic agents and insulin treatments (18). A Norway study (13) of 9 CEL-MODY patients revealed pancreatic enzyme replacement therapy improved the steatorrhea and elevated levels of fat-soluble vitamins E and A for 30-month follow-up. However, fecal elastase levels did not change significantly. The incidence of diabetic complications of CEL-MODY is extremely low. One CEL-MODY patient died from renal failure at age 65 years old (2). The trigger of renal failure was not mentioned. In this study, the patient’s glycemic levels have remained within normal ranges with dietary control. There were no signs of pancreatic atrophy and steatosis. The progression of the disease should be tracked by detailed follow-up.

## Conclusions

We described a 12-year-old boy carrying a novel mutation in the *CEL* gene causing the mild phenotype. It may be due to mutation position and age of onset. For pediatric patients carrying *CEL* gene mutations should be followed up and monitored for a long time. Our case highlighted the heterogeneity of the *CEL* gene mutations and its various phenotypes in children. The inadequacy of our study is the lack of functional experiments to further investigate the mutation’s pathogenicity and verify the length of the VNTR region. Future research focusing on the relation of phenotypes and genotypes associated with *CEL* gene mutations remains necessary.

## Data Availability

The datasets presented in this study can be found in online repositories. The names of the repository/repositories and accession number(s) can be found in the article/supplementary material.
